# Intraoperative near-infrared fluorescence imaging can identify pelvic nerves in patients with cervical cancer in real time during radical hysterectomy

**DOI:** 10.1007/s00259-022-05686-z

**Published:** 2022-03-01

**Authors:** Kunshan He, Pengfei Li, Zeyu Zhang, Jiaqi Liu, Pan Liu, Shipeng Gong, Chongwei Chi, Ping Liu, Chunlin Chen, Jie Tian

**Affiliations:** 1 Beijing Advanced Innovation Center for Big Data-Based Precision Medicine, School of Engineering Medicine, Beihang University, Beijing, 100191 China; 2 Key Laboratory of Big Data-Based Precision Medicine (Beihang University), Ministry of Industry and Information Technology, Beijing, 100191 China; 3 CAS Key Laboratory of Molecular Imaging, The State Key Laboratory of Management and Control for Complex Systems, Institute of Automation, Chinese Academy of Sciences, Beijing, 100190 China; 4 State Key Laboratory of Computer Science and Beijing Key Lab of Human-Computer Interaction, Institute of Software, Chinese Academy of Sciences, Beijing, China; 5 Department of Obstetrics and Gynecology, Nanfang Hospital, Southern Medical University, Guangzhou, China

**Keywords:** Near-infrared fluorescence imaging, Indocyanine green, Pelvic nerves, Radical hysterectomy, Cervical cancer

## Abstract

**Purpose:**

Radical hysterectomy combined with pelvic lymphadenectomy is the standard treatment for early-stage cervical cancer, but unrecognized pelvic nerves are vulnerable to irreversible damage during surgery. This early clinical trial investigated the feasibility and safety of intraoperative near-infrared (NIR) fluorescence imaging (NIR-FI) with indocyanine green (ICG) for identifying pelvic nerves during radical hysterectomy for cervical cancer.

**Methods:**

Sixty-six adults with cervical cancer were enrolled in this prospective, open-label, single-arm, single-center clinical trial. NIR-FI was performed in vivo to identify genitofemoral (GN), obturator (ON), and hypogastric (HN) nerves intraoperatively. The primary endpoint was the presence of fluorescence in pelvic nerves. Secondary endpoints were the ICG distribution in a nerve specimen and potential underlying causes of fluorescence emission in pelvic nerves.

**Results:**

In total, 63 patients were analyzed. The ON was visualized bilaterally in 100% (63/63) of patients, with a mean fluorescence signal-to-background ratio (SBR) of 5.3±2.1. The GN was identified bilaterally in 93.7% (59/63) of patients and unilaterally in the remaining 4 patients, with a mean SBR of 4.1±1.9. The HN was identified bilaterally in 81.0% (51/63) of patients and unilaterally in 7.9% (5/63) of patients, with a mean SBR of 3.5±1.3. ICG fluorescence was detected in frozen sections of a nerve specimen, and was mainly distributed in axons. No ICG-related complications were observed.

**Conclusion:**

This early clinical trial demonstrated the feasibility and safety of NIR-FI to visualize pelvic nerves intraoperatively. Thus, NIR-FI may help surgeons adjust surgical decision-making, avoid nerve damage, and improve surgical outcomes.

**Trial registration:**

ClinicalTrials.gov NCT04224467

## Introduction

Cervical cancer is the fourth most common cancer in women worldwide [1]. The 5-year survival rate of stage III or IV cervical cancer is less than 30%, and its incidence tends to occur in younger ages in recent years [2]. Radical hysterectomy combined with pelvic lymphadenectomy is one of the most effective treatments for cervical cancer [3]. However, as the pelvic nerves are difficult to recognize and are vulnerable, the surgical outcome is limited by irreversible neural damage [4].

Unfortunately, damage to the pelvic nerves can result in severe motor, sensory, and autonomic dysfunction. As reported previously, the incidence of genitofemoral nerve (GN) damage is 3.5%, and causes paresthesia in the groin and perineum [5]. The incidence of obturator nerve (ON) injury is 1%, which may reduce thigh adduction and induce slight abnormal sensations in the inner thigh skin [6]. Furthermore, 30–80% of patients undergoing traditional radical hysterectomy have bladder, rectal, or sexual dysfunction, mainly caused by pelvic autonomic nerve injury [4]. Intraoperative nerve visualization is considered crucial to reducing pelvic nerve damage [7]. Clinically, the identification of pelvic nerves mainly depends on visual inspection, which relies heavily on the surgeon’s experience [8]. Methods such as nerve stimulation have been employed to identify nerves; however, the procedure is complex and the effect is limited. Consequently, there has been a growing desire to develop novel nerve visualization methods [9, 10].

Currently, emerging near-infrared (NIR) fluorescence imaging (NIR-FI) is playing an increasingly important role in surgery [11, 12]. Because of its increased penetration depth, lower autofluorescence, and higher sensitivity, NIR-FI has been applied in tumor detection, lymphangiography, and vascular perfusion assessment [13–16]. Clinical observations have also revealed its potential to image nerves intraoperatively. Using widely employed fluorophore indocyanine green (ICG), we previously reported the feasibility of intraoperative NIR-FI for distinguishing thoracic sympathetic nerves in 15 patients with lung cancer [17].

Based on these findings, we hypothesized that intraoperative NIR-FI with ICG could be a new method for real-time identification of pelvic nerves during surgery. This early clinical trial investigated the feasibility and safety of intraoperative NIR-FI for identifying pelvic nerves during radical hysterectomy in patients with cervical cancer.

## Methods

### Study design

This prospective, open-label, single-arm clinical trial was approved by the Medical Ethics Committee of Nanfang Hospital of Southern Medical University (Ethics no. NFEC-2019-146), and registered at ClinicalTrials.gov (NCT04224467). The primary objective of this early clinical trial was to investigate the feasibility and safety of NIR-FI with ICG for identifying the pelvic nerves. Secondary endpoints were the distribution of ICG in a nerve specimen and potential underlying causes of fluorescence emission in pelvic nerves. Sixty-six patients with cervical cancer were enrolled and provided written informed consent before inclusion between February 2020 and June 2021. The inclusion criteria were as follows: cervical cancer with preoperative FIGO staging IB1-IIA2 (FIGO 2018 staging system), age >18 years, and scheduled for radical hysterectomy combined with pelvic lymphadenectomy. The exclusion criteria were as follows: a history of pelvic surgery, radiotherapy, or chemotherapy; pregnancy; and considered unsuitable for enrollment by clinicians.

### Contrast agent

ICG (25 mg per vial) was purchased from Dandong Yichuang Pharmaceutical Co. Ltd., China. ICG has been approved for clinical use for more than 60 years, with good safety and fluorescence performance (peak absorption/emission, 800/830 nm) [18]. Each vial of ICG was dissolved in its own 10 ml injectable water as the stock solution. After an allergy test, intravenous infusion of ICG (5 mg/kg) diluted in 500 ml saline was administered 24 h prior to surgery based on our previous study of thoracic sympathetic nerves NIR-FI [17].

### Fluorescence imaging

NIR-FI with ICG in vivo was performed using a DPM-I system (Zhuhai Dipu Medical Technology Co., Ltd.) developed for open surgery, in accordance with our previous studies [19, 20]. In brief, DPM-I consists of a hybrid light source generating two wavelength-isolated lights: 1200 lm of 420–700 nm light and 35 mW/cm^2^ of 785 nm light. The fluorescence and color images were simultaneously acquired using custom optics and software at a work distance of 15–30 cm. Color images, fluorescence images, or merged images, or all three images together can be displayed as needed, with a resolution of 1280×1024. The depth of the imaging object from the surface should be within 10 mm. During pelvic lymphadenectomy, when the external iliac lymph nodes were removed, NIR-FI was used to visualize the GN on the lateral side of the external iliac artery and the surface of the psoas muscle. Similarly, the ON was visualized in the obturator space when the obturator lymph node was resected. During the radical hysterectomy, when the vaginal and lateral rectal spaces were opened, and the ureteral mesentery was exposed, the hypogastric nerve (HN) was visualized by fluorescence.

### Histopathologic analysis

In one patient, the HN was accidentally cut off during exposure to the mesoureter. With the patient’s consent, the nerve was used for histopathological analysis. Frozen transverse and longitudinal sections of the nerve (thickness, 10 mm) were prepared in a standard fashion and stored at −20°C for pathological analysis. Anti-myelin basic protein (MBP) antibody (1:200; ABN912, Sigma, St. Louis, MO, USA) was used to label the myelin sheath, rabbit anti-NF 200 antibody (1:800; N4142, Sigma, St. Louis, MO, USA) was used to label axons, and DAPI (1:5,000; Sigma, St. Louis, MO, USA) was used for nuclear staining. Fluorescence microscopy was performed using a laser confocal microscope (LSM980, Zeiss, Germany).

### Cell tests

Human umbilical vein endothelial cells (HUVECs), vascular smooth muscle cells (VSMCs), and hepatic cells (HL-7702) were purchased from the cell bank of the Chinese Academy of Sciences (Shanghai, China) and cultured in DMEM medium containing 10% fetal bovine serum (FBS) and 1% penicillin-streptomycin solution (Gibco) in a humidified incubator (5% CO_2_ at 37°C). Neurons and Schwann cells were obtained by primary cell culture, as described by Wen et al. [21].

### ICG transport test

The uptake and secretion of ICG were compared between neurons, Schwann cells, HUVECs, VSMCs, and hepatic cells. Briefly, the cells in 12-well plates (counted as 2×10^5^ cells/well) were incubated with 0.5 mg/ml ICG-containing medium. After incubation for 5 min, the cells were washed four times with 1 ml of phosphate buffered saline (PBS) and subsequently lysed with 300 μl of 1% Triton-X100 in PBS. Concentrations of ICG uptake were measured with a spectrophotometer (Varioskan Flash, Thermo Fisher Scientific) at 805 nm, using a standard curve. For the ICG secretion test, after incubation with ICG (0.5 mg/ml) for 5 min, the cells were washed four times with 1 ml of PBS; the medium was then replaced with DMEM containing 10% FBS and 1% penicillin-streptomycin solution. After incubation for 12 h, intracellular residual ICG concentrations were measured as described above.

### Statistical analysis

The specific fluorescence of each identified pelvic nerve in vivo was analyzed based on intraoperatively recorded images. For each case, the fluorescence signal-to-background ratio (SBR) of the pelvic nerves relative to adjacent tissues was calculated three times to reduce deviation. First, regions of interest (ROIs) were delineated over the fluorescent pelvic nerves and surrounding tissue using ImageJ software (National Institutes of Health, USA). For each ROI, the mean fluorescence intensity was expressed within a range of 0–255, allowing the calculation of the SBR.

All statistical analyses were performed using SPSS 23.0 statistical software (Statistical Product and Service Solutions, Chicago, USA). Categorical variables are expressed as whole numbers and percentages, and were compared using the chi-square test. Continuous variables are expressed as mean ± standard deviation. One-way analysis of variance was used to compare SBRs among nerves. Multiple linear regression was used to estimate the impact of clinicopathological characteristics on the SBR of the ON, GN, and HN, including age, height, weight, preoperative FIGO stage, histology, differentiation, stromal invasion, lymphovascular space invasion (LVSI), parametrial tumor involvement, and lymphatic metastasis. *P*-values of 0.05 or less were considered statistically significant.

## Results

### Patient characteristics

Sixty-six consecutive patients were initially enrolled in this study. However, two patients temporarily refused intravenous ICG therapy before the surgery. Additionally, one patient was intraoperatively diagnosed with suspected lymph node metastasis, which was confirmed by frozen pathology after lymph node biopsy; systematic pelvic lymphadenectomy and radical hysterectomy were not performed. Thus, 63 patients were analyzed. Their clinicopathologic characteristics are shown in Table 1. No ICG-related adverse events occurred in any patient.

**Table 1:** The clinicopathologic characteristics of patients (*n*=63)

Characteristics	Value	Percentage
Age (years)	49.41±10.317	
Histologic type		
Squamous cell	51	80.95
Adenocarcinoma	9	14.29
Adenosquamous	3	4.76
Cervical conization		
No	53	84.13
Yes	10	15.87
Preoperative FIGO stage		
IB1	24	38.10
IB2	13	20.63
IB3	4	6.35
IIA1	16	25.40
IIA2	6	9.52
Final FIGO stage		
IB1	18	28.57
IB2	8	12.70
IB3	3	4.76
IIA1	14	22.22
IIA2	3	4.76
IIB	2	3.17
IIC1P	15	23.81
Stromal invasion		
Superficial	22	34.92
Deep	41	65.08
Lymphovascular space invasion		
Negative	49	77.78
Positive	14	22.22
Parametrium		
Negative	61	96.83
Positive	2	3.17
Surgical margin		
Negative	61	96.83
Positive	2	3.17
Pelvic lymph nodes metastasis		
Negative	48	76.19
Positive	15	23.81

*FIGO*, International Federation of Gynecology and Obstetrics

### Pelvic nerve NIR-FI

**Fig. 1:**
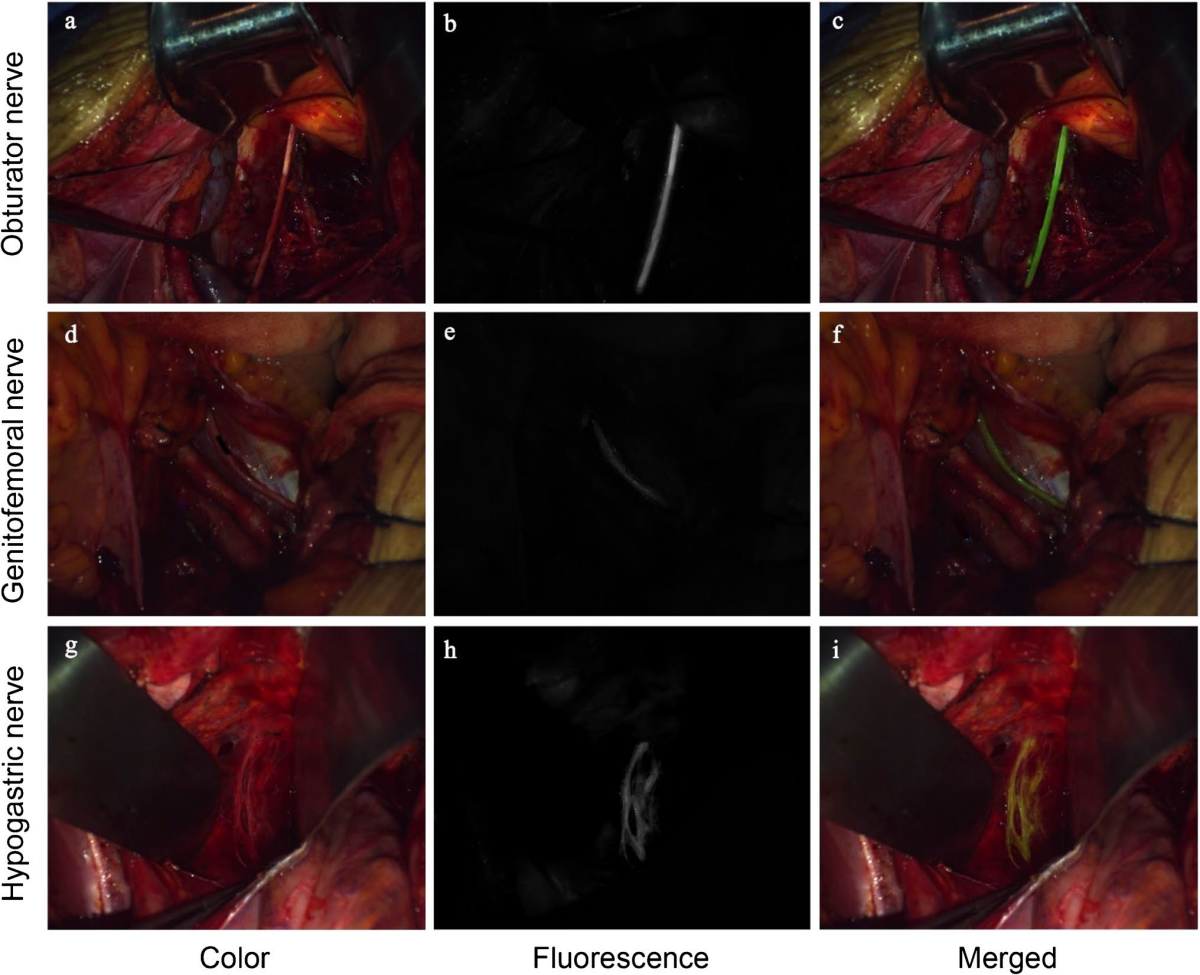
Representative images of the **a**–**c** obturator, **d**–**f** genitofemoral, and **e**–**h** hypogastric nerves

Using DPM-I with ICG, the ON was successfully identified bilaterally (Fig. 1a–c) in all patients, and the mean SBR of the ON was 5.3±2.1 (Table 2). The GN (Fig. 1d–f) was identified bilaterally in 93.7% (59/63) of patients and unilaterally in the remaining 4 patients. The mean SBR of the GN was 4.1±1.9. The HN (Fig. 1g–i) was identified bilaterally in 81.0% (51/63) of patients and unilaterally in 7.9% (5/63) of patients; the HN was not identified in 11.1% (7/63) of patients. The mean SBR of the HN was 3.5±1.3. The above three SBR values are high enough to assist surgeons in identifying the corresponding nerves during surgery, and there was no statistical difference among them (*p*=0.13). Moreover, a strong fluorescent signal lasted until the end of the operation in all cases, and no obvious intensity changes were observed during the entire operation. The longest observed fluorescence duration was 245 min.

**Table 2:** The mean signal-to-background ratio of pelvic nerves

Pelvic nerves	SBR	*P* value
Obturator nerve	5.3±2.1	
Genitofemoral nerve	4.1±1.9	0.13
Hypogastric nerve	3.5±1.3	

On multiple linear regression, the SBR of the OB, ON, and HN was not related to the preoperative FIGO stage, age, weight, height, histology, differentiation, stromal invasion, LVSI, surgical margin invasion, parametrial tumor involvement, or lymph node metastasis (all *p*>0.05).

### Pathologic findings

**Fig. 2:**
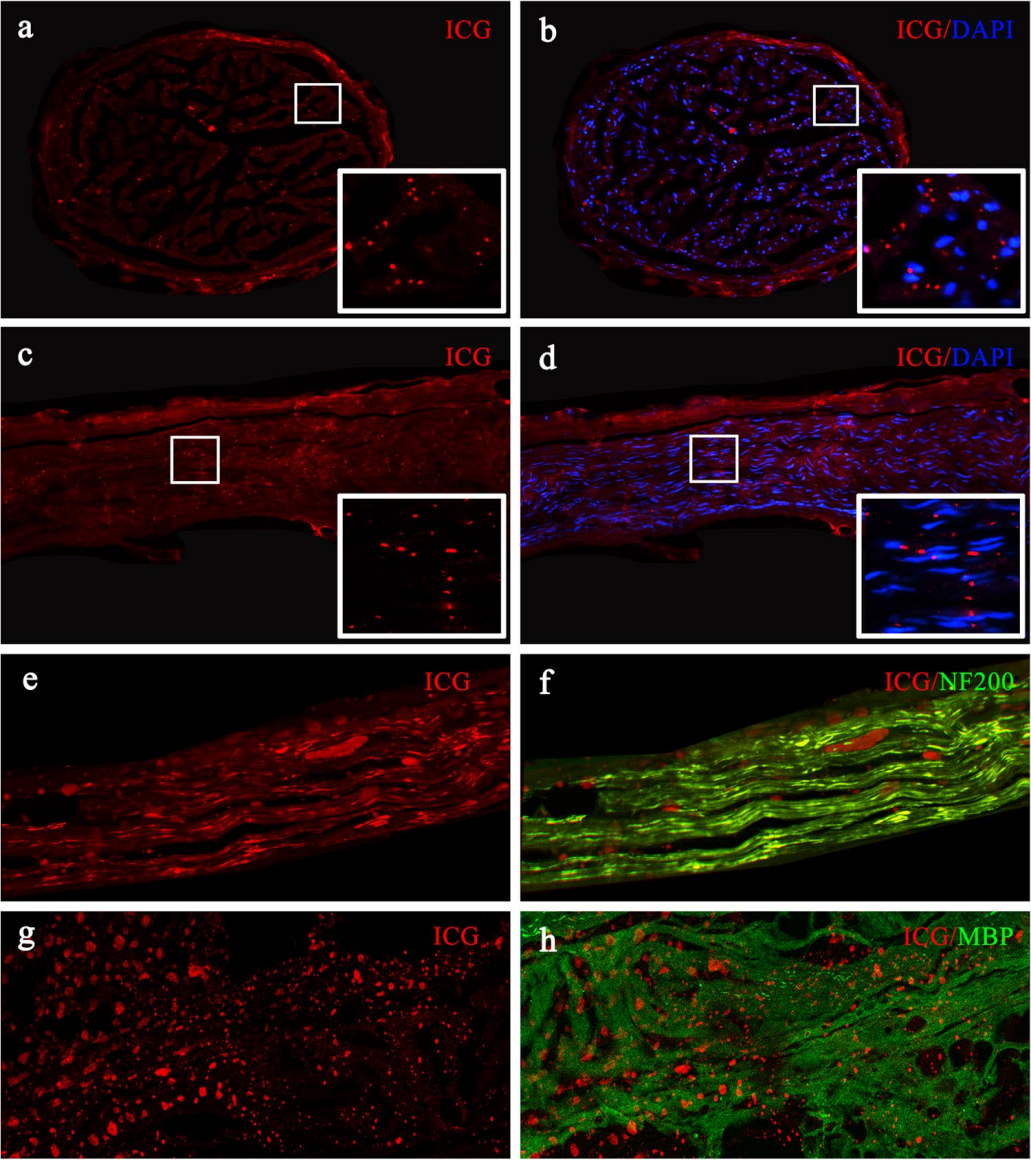
Fluorescent microscopy confirmed that ICG was mainly distributed in the axon of the hypogastric nerve. **a**–**b** The transverse and **c**–**d** longitudinal sections of the hypogastric nerve; **e**–**f** ICG fluorescent signal distributed in axons labeled with anti-NF 200 antibody; and **g**–**h** less ICG fluorescence signal in myelin sheath labeled with anti-MBP antibody

Fluorescent microscopy confirmed the presence of ICG fluorescent signal in the HN specimen, with fluorescence signals mainly in the cytoplasm rather than in the nucleus (Fig. 2a–d). Confocal laser scanning microscopy (CLSM) images revealed that the red ICG fluorescence overlapped with the green fluorescence of axons labeled with anti-NF 200 antibody (Fig. 2e–f), but showed little overlap with myelin sheath labeled by anti-MBP antibody (Fig. 2g–h). In other words, the ICG fluorescent signal was mainly distributed in the axons of the nerve specimen.

### Cell test findings

**Fig. 3:**
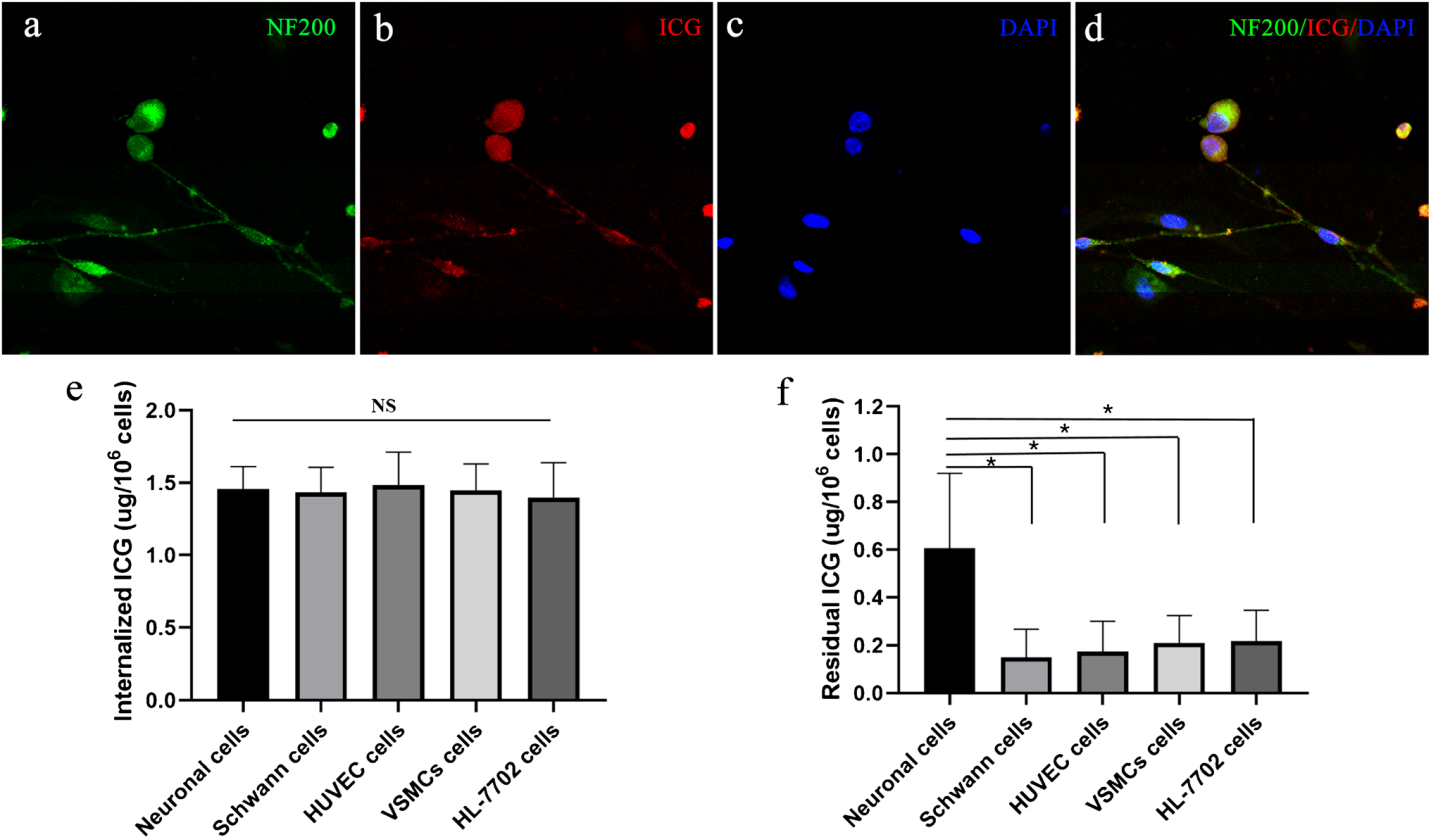
ICG uptake and secretion cell test. **a**–**d** ICG can be taken up by neuronal cells. **e** The internalized ICG of different cells is similar after 5 min of incubation with an ICG-containing medium. **f** Neuronal cells secreted ICG significantly slower than other cells after incubation with 0.5 mg/ml ICG for 5 min and DMEM medium for 12h

After incubation with an ICG-containing medium for 5 min, ICG fluorescent signals were detected in all cells on CLSM images. Figure 3a–d shows that ICG can be absorbed by neuronal cells. The internalized ICG of neurons, Schwann cells, HUVECs, VSMCs, and HL-7702 cells was 1.46±0.15, 1.43±0.17, 1.48±0.23, 1.45±0.18, and 1.39±0.24 μg/10^6^ cells, respectively. The internalized ICG did not significantly differ among cell types (*p*=0.97), as shown in Fig. 3e. In the ICG secretion test, after incubation with ICG (0.5 mg/ml) for 5 min and DMEM medium for 12 h, the residual ICG in neuronal cells was 0.61±0.31 μg/10^6^ cells, which was significantly higher than that in other cell types (*p*=0.003), as shown in Fig. 3f.

## Discussion

Here, we investigated the feasibility and safety of intraoperative NIR-FI using the DPM-I system with ICG to identify the pelvic nerves in a prospective trial. To the best of our knowledge, this is the first clinical trial of intraoperative NIR-FI with ICG for identifying the pelvic nerves during radical hysterectomy for cervical cancer. The bilateral identification rates of the ON, GN, and HN were 100% (63/63), 93.7% (59/63), and 81.0% (51/63), respectively. Pathological results and cell tests confirmed our findings, suggesting that this may be a new method for identifying pelvic nerves that may significantly reduce functional damage and improve surgical outcomes.

Although a cure is the main goal of surgery, the protection of important structures, such as nerves, is also critical to achieving the best patient prognosis [22, 23]. Iatrogenic nerve injury is one of the most frightening complications in surgical procedures, as well as a major source of morbidity in all surgical specialties [24]. In patients with cervical cancer undergoing radical hysterectomy combined with pelvic lymphadenectomy, extensive removal of the peri-cervix ligaments (uterosacral, main, and bladder-cervical ligaments, and paravaginal tissue) and para-aortic fibrous connective tissue is commonly applied [23]. Therefore, the pelvic nerves, including the ON, GN, and HN, are vulnerable to damage, which may cause bladder, anorectal, and sexual dysfunction [6, 25]. More seriously, approximately 50% of patients with these symptoms will suffer for life.

Because most nerves are relatively thin and located under the surrounding tissues, it is difficult for even experienced surgeons to identify them with the naked eye during surgery [26]. Furthermore, previous radiotherapy or surgery may lead to fibrotic tissue deposition and atypical surgical anatomical planes, rendering nerve recognition more challenging [24]. Currently, in some surgical procedures, surgeons use optical coherence tomography, Raman spectroscopy, or confocal laser endomicroscopy to help identify nerves. However, these methods lack wide­field imaging capabilities. Intraoperative ultrasound has also shown potential in superficial nerve visualization, but its low resolution and lack of specificity hinder its clinical application. Electromyography, which involves placing electrodes in the muscle innervated by the nerve of interest, is currently the most commonly used technique for intraoperative peripheral nerve identification. However, due to its many restrictions and time-consuming and labor-intensive use, it is rarely used in cervical cancer surgery at present. In summary, there is an urgent need to develop a new method for identifying nerves during surgery, with the goal of preventing accidental injury.

Fluorescence-guided surgery has shown great potential in pre-clinical research and clinical applications for identifying lesions and important functional tissues (such as nerves). In particular, fluorophores in NIR spectroscopy have aroused great interest from researchers. In this spectrum, tissue autofluorescence, photon absorption, and scattering fall to local minima, thereby achieving millimeter-level resolution and centimeter-level penetration depth. Non-specific fluorophores can highlight nerves through diffusion, active reuptake at synapses, or accumulate in adjective tissues. For example, Samarasena et al. used NeuroTrace to visualize enteric nerves located in the gastric mucosa in an animal model [27]. In contrast, targeted fluorophores improve the SBR by binding to specific moieties or proteins in nerves. Hingorani et al. synthesized FAM-HNP401 and measured the nerve-to-muscle contrast after its topical or systemic administration in pre-clinical trials [28]. However, the research and exploration of nerve NIR-FI are still in their infancy; especially, its application in clinical surgery for gynaecologic tumors is very rare.

In the present study, we found that NIR-FI with ICG could effectively identify the ON, GN, and HN. Although the HN was not identified in seven patients, this early clinical trial showed that the pelvic nerve NIR-FI results did not significantly correlate with the patient’s age, height, weight, preoperative FIGO stage, histology, differentiation, stromal invasion, LVSI, parametrial tumor involvement, or lymph node metastasis. The lower identification rate for the HN may be related to the size of the nerve, as it is thinner than the ON and GN. Furthermore, in the cell tests, the uptake of ICG by neuronal cells was similar to that of other cells. However, neuron cells excrete ICG more slowly, leading to concentration differences within a certain period of time, which may be related to the axonal transport of ICG in neurons; this may be the cause of fluorescence emission in pelvic nerves under NIR-FI. Therefore, the ICG injection dose, timing, and sensitivity of NIR-FI devices are closely related to the imaging results.

We acknowledge that there are several limitations to this study. First, this was an early clinical trial, and its main purpose was to investigate the feasibility and safety of NIR-FI for identifying pelvic nerves. Therefore, it was designed as a single-arm study. We did not conduct a randomized controlled trial to compare nerve identification and injury rates between NIR-FI-guided surgery and traditional surgery. Second, we analyzed the NIR-FI of only the HN, not all pelvic autonomic nerves, such as the superior hypogastric plexus, pelvic splanchnic nerves, and inferior hypogastric plexus. Because the HN is easy to locate, it is suitable as an objective and reliable index of imaging results. Third, the follow-up time was too short to evaluate the relationship between the results of pelvic nerve NIR-FI and the patient’s prognosis. Finally, ICG is not a nerve-targeted contrast agent. The development of new nerve-targeted contrast agents may provide better imaging results.

In conclusion, these results indicate that intraoperative NIR-FI using ICG can effectively and safely identify pelvic nerves in patients with cervical cancer in real time. The NIR-FI of nerves has the potential to reduce functional impairment and help surgeons adjust decision-making. This research lays the foundation for further clinical pelvic nerve NIR-FI trials, including the use of specific fluorophores, if available.

## Data Availability

The datasets generated during and/or analyzed during the current study are not publicly available due to the use of patient data but are available from the corresponding author on reasonable request.
